# A one step real-time RT-PCR assay for the quantitation of *Wheat yellow mosaic virus* (WYMV)

**DOI:** 10.1186/1743-422X-10-173

**Published:** 2013-06-01

**Authors:** Wenwen Liu, Xiaojuan Zhao, Peng Zhang, Thi Thi Mar, Yan Liu, Zongying Zhang, Chenggui Han, Xifeng Wang

**Affiliations:** 1 State Key Laboratory for Biology of Plant Diseases and Insect Pests, Institute of Plant Protection, Chinese Academy of Agricultural Sciences, No. 2, West Yuan Ming Yuan Road, Beijing 100193, China; 2 Department of Plant Pathology and State Key Laboratory for Agrobiotechnology, China Agricultural University, Beijing 100193, China

**Keywords:** *Wheat yellow mosaic virus* (WYMV), RT-qPCR, Quantitation

## Abstract

**Background:**

*Wheat yellow mosaic virus* (WYMV) is an important pathogen in China and other countries. It is the member of the genus *Bymovirus* and transmitted primarily by *Polymyxa graminis*. The incidence of wheat infections in endemic areas has risen in recent years. Prompt and dependable identification of WYMV is a critical component of response to suspect cases.

**Methods:**

In this study, a one step real-time RT-PCR, followed by standard curve analysis for the detection and identification of WYMV, was developed. Two reference genes, 18s RNA and β-actin were selected in order to adjust the veracity of the real-time RT-PCR assay.

**Results:**

We developed a one-step Taqman-based real-time quantitative RT-PCR (RT-qPCR) assay targeting the conserved region of the 879 bp long full-length WYMV coat protein gene. The accuracy of normalized data was analyzed along with appropriate internal control genes: β-actin and 18s rRNA which were included in detecting of WYMV-infected wheat leaf tissues. The detectable end point sensitivity in RT-qPCR assay was reaching the minimum limit of the quantitative assay and the measurable copy numbers were about 30 at10^6^-fold dilution of total RNA. This value was close to 10^4^-fold more sensitive than that of indirect enzyme-linked immunosorbent assay. More positive samples were detected by RT-qPCR assay than gel-based RT-PCR when detecting the suspected samples collected from 8 regions of China. Based on presented results, RT-qPCR will provide a valuable method for the quantitative detection of WYMV.

**Conclusions:**

The Taqman-based RT-qPCR assay is a faster, simpler, more sensitive and less expensive procedure for detection and quantification of WYMV than other currently used methods.

## Background

More than 50 different viruses are known to infect wheat (*Triticum aestivum*) worldwide, causing severe symptoms which can affect negatively the yield and quality production [1]. In China, several virus species have been reported to infect cereals: *Barley yellow dwarf virus* (BYDV)/*Cereal yellow dwarf virus* (CYDV) (genus *Luteovirus*/*Polerovirus*), *Wheat yellow mosaic virus* (WYMV) (genus *Bymovirus*), *North cereal mosaic virus* (NCMV) (genus *Cytorhabdovirus*) and *Wheat dwarf virus* (WDV) (genus *Mastrevirus*) [2-4]. They are transmitted by different species of aphids, planthoppers or the fungus-like organism *Polymyxa graminis* in a persistent manner [1,2]. Statistical data showed that the disease area was more than 6000,000 ha in the 2000s, and the yield loss was estimated within the range 10–20% and up to 40–50% in some years with serious occurrence [2].

Since the mid-1970s, wheat yellow mosaic disease has been reported in different regions of China, especially in the middle and lower reaches of the Yangtze River, the Sichuan basin, the wheat belt along the Huaihe River, and the Weihe River basin of Shanxi Province [5,6]. Filamentous viruses transmitted by *P. graminis* cause similar diseases of wheat in Europe, Asia and North America [7,8]. These viruses have been described as *Wheat spindle streak mosaic virus* (WSSMV) and WYMV in different countries [7,9,10]. Until now, the virus causing wheat soil-borne mosaic diseases has been identified as WYMV in most areas of China [11,12]. The genome of WYMV is comprised of two (+) single-stranded RNAs. RNA1 encodes for the coat protein (CP) and six others: P3, 7 KDa, nuclear inclusion protein a (NIa), nuclear inclusion protein b (NIb), cytoplasmic inclusion protein (CI) and 14 KDa; RNA2 encodes for a polyprotein that contains 28- and 72-kDa proteins [13,14].

Symptoms of WYMV infection are similar to those caused by WSSMV and other biotic and abiotic agents. For this reason and the fact that several virus species exist together in the field, detection and diagnosis of WYMV is an important area of study. Several diagnostic methods are available for WYMV detection in wheat plants, including biological assay (virus inoculation and symptom expression in diagnostic plant species), ELISA (enzyme-linked immunosorbent assay) [15], immunoelectron microscopy, RT-PCR (reverse transcription polymerase chain reaction; [16] and RT-LAMP (reverse transcription loop-mediated isothermal amplification; [17] However, biological assays are time-consuming and have low levels of sensitivity and specificity. ELISA and immunoelectron microscopy are limited by the supply and quality of antiserum or the specific probe, as well as by the type of sampling. Because information on the virus titre in local fields is very important for forecasting and releasing warning schemes to advise farmers on the potential threat to their crops, a sensitive, reliable and quantitative method is required to detect WYMV in wheat. Nowadays, real-time PCR technology has been proven as an efficient tool for the detection of many plant RNA and DNA viruses [18-24]. This work presents a new real time RT-PCR assay for the detection of WYMV in infected plant tissue. The assay’s sensitivity was investigated and compared with indirect ELISA and evaluated for virus detection in field samples.

## Result

### Optimization of RT-qPCR

**Figure 1:**
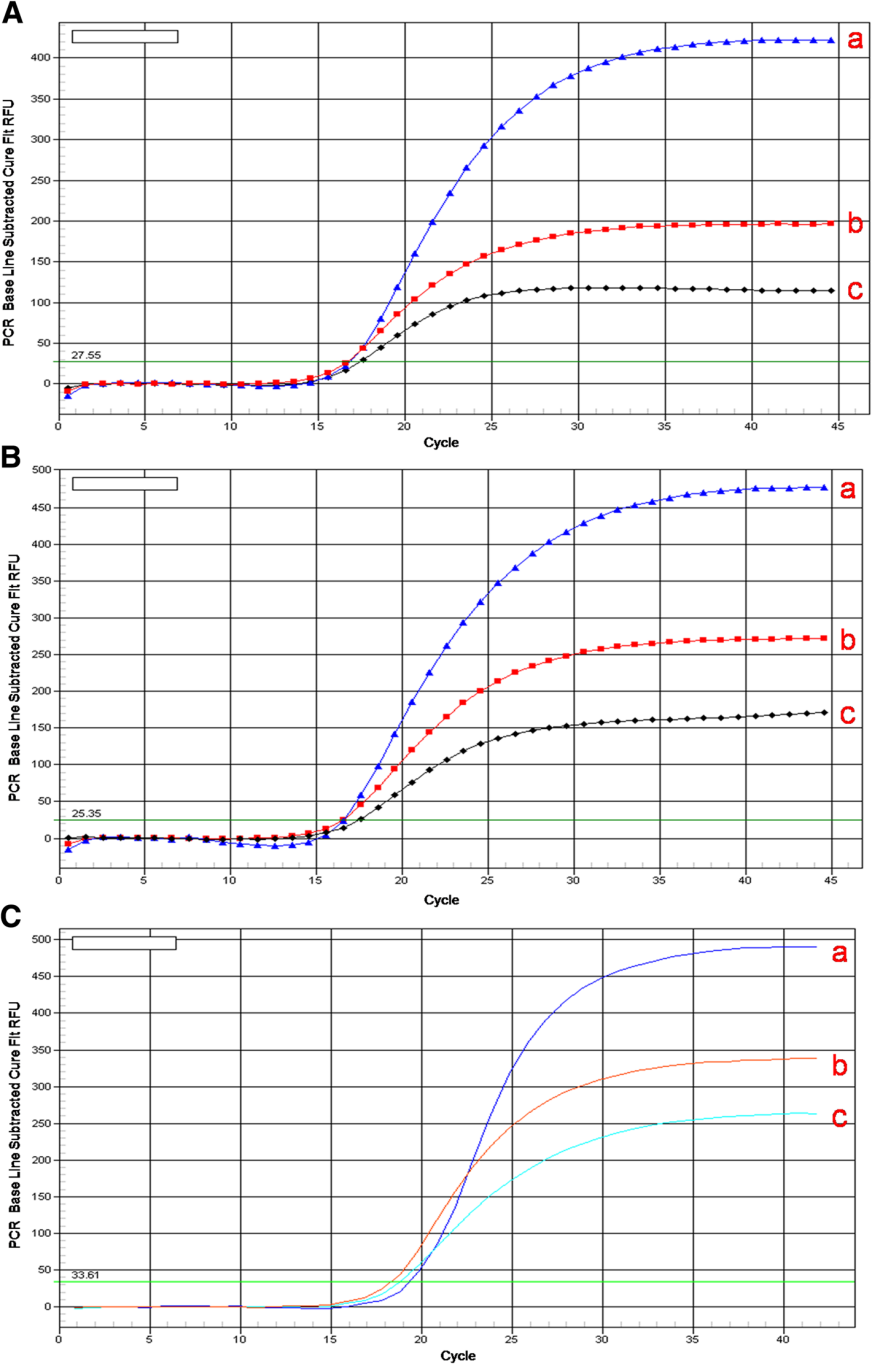
**Optimization concentration of primer and probe: A: standard sample of WYMV as template. concentration of upstream, downstream primer and probe at a)200 nM 200 nM 200 nM; b) 200 nM 200 nM 400 nM; c) 100 nM 100 nM 200 nM. B**: standard sample of β-actin as template. concentration of upstream, downstream primer and probe at a)200 nM 200 nM 200 nM; b) 200 nM 200 nM 400 nM; c) 100 nM 100 nM 200 nM. **C**: standard sample of 18S rRNA as template. concentration of upstream, downstream primer and probe at a)200 nM 200 nM 200 nM; b) 200 nM 200 nM 400 nM; c) 100 nM 100 nM 200 nM.

The optimum concentration for both upstream and downstream primers of WYMV was found to be 200 nM. Detection of the WYMV, wheat β-actin and 18s rRNA targeted by real-time RT-PCR was efficient and reproducible with 200 nM TaqMan probe (Figure 1).

### Standard curves of RT-qPCR

**Figure 2:**
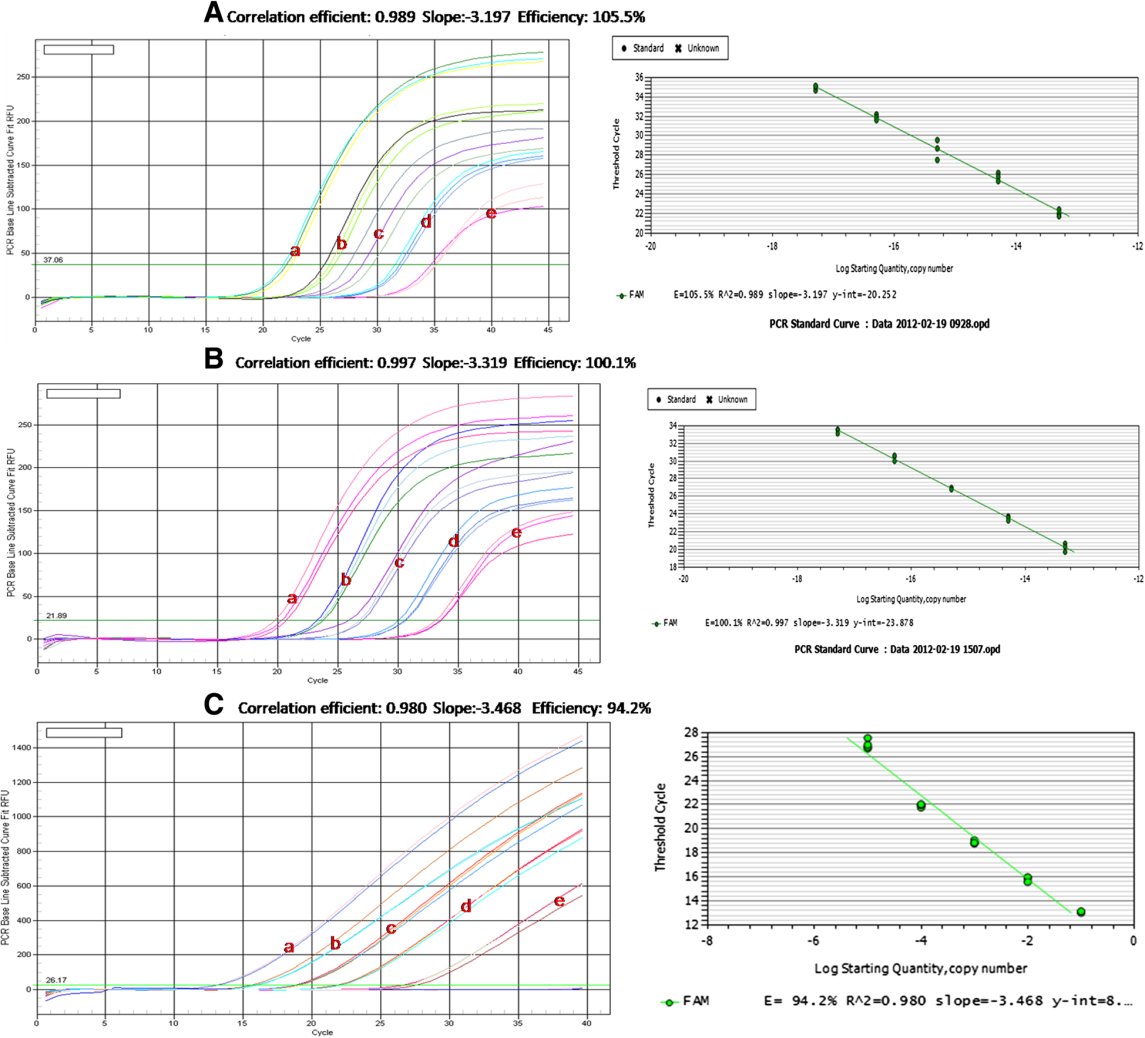
**Standard curve for TaqMan real time RT-PCR amplification of standard ssRNA (viral transcripts): A: Amplification plots showing the testing in triplicate of a 10-fold dilution series containing standard ssRNA of WYMV at: 2x10**^**5**^**(a); 2x10**^**4**^**(b); 2x10**^**3 **^**(c); 2x10**^**2**^**(d); 2x10**^**1**^**(e) template copies per reaction. B**: Amplification plots showing the testing in triplicate of a 10-fold dilution series containing standard ssRNA of WYMV at: 1x10^6^(a); 1x10^5^(b); 1x10^4^ (c); 1x10^3^(d); 1x10^2^(e) template copies per reaction. **C**: Amplification plots showing the testing in triplicate of a 10-fold dilution series containing standard ssRNA of WYMV at: 2x10^5^(a); 2x10^4^(b); 2x10^3^ (c); 2x10^2^(d); 2x10^1^(e) template copies per reaction.

The linear range of quantitation of the one-step real-time RT-PCR assay for WYMV genomic RNA was determined by using tenfold serial dilutions of the standard ssRNA ranging from 20 to 2 × 10^5^ copies to determine the end-point limit of detection and the linearity of the assay. Ct-values were measured in three duplicates and plotted against the known copy numbers of the standard sample. The standard curve (Figure 2A) covered a linear range of five orders of magnitude. The slope (−3.197) and the correlation coefficient (*R*^2^ = 0.989) of the standard curve showed that this assay could be used to quantify target RNA in infected wheat tissue. Dilution curves were obtained with total RNA from WYMV-infected wheat and their amplification efficiency was similar to that of the standard curves. This real-time RT-PCR assay enabled to detect as few as 20 copies of the WYMV CP gene in wheat total RNA extracts (Figure 2A).

A standard curve of quantification of β-actin gene (Figure 2B) and 18s rRNA (Figure 2C) gene in wheat were developed respectively as described above. According to the differences of original standard sample concentration, different copy ranges were used to determine the standard curves as follow: The linear ranges of β-actin gene and 18s rRNA gene were 106 to 102 copies, –3.319 and −3.303, and R^2^ = 0.997 and 0.919, respectively.

### Comparison of sensitivity between indirect ELISA and real time RT-PCR

In order to compare the end point sensitivity between TaqMan RT-qPCR and ELISA assay, ten-fold dilutions of crude sap obtained from infected wheat leaf were prepared. In indirect ELISA assay, a positive reaction (twice that of the negative control) was only recorded from tenfold dilution of the crude sap from 100 mg virus-infected leaf tissues. The *A*_405_ values of 10 fold dilution samples and the negative control were 0.79, and 0.041, respectively. Simultaneously, RT-qPCR assay was performed with ten-fold serial dilution of total RNA from 100 mg infected leaf tissue to detect end point sensitivity. The detectable end point sensitivity was reaching the minimum limit of the quantitative assay and the measurable gene copy numbers were about 30 at10^6^-fold dilution of total RNA (Table 1). These results indicated that the dilution limit for indirect ELISA was tenfold, while WYMV genomic RNA was easily detectable at a corresponding dilution by real time RT-PCR assay. The results showed that real-time RT-PCR was nearly 10^4^-fold more sensitive than indirect ELISA.

**Table 1:** Detection of WYMV in tenfold serial dilutions of the crude sap from infected wheat by indirect ELISA and real-time RT-PCR

	**Negative control**^**a**^	**Positive control**^**b**^	**1/10th**	**1/10**^**2**^**th**	**1/10**^**3**^**th**	**1/10**^**4**^**th**	**1/10**^**5**^**th**	**1/10**^**6**^**th**
Mean of A_405_^c^	0.041	1.093	0.79	0.073	0.052	0.013	0.012	0.008
Number of WYMV genomic RNA copies	–	3.09×10^7^	2.99×10^6^	3.11×10^5^	3.10×10^4^	2.91×10^3^	3.15×10^2^	30

^a^ Healthy wheat leaf; ^b^ WYMV-infected wheat leaf; ^c^ Mean calculated using all replications.

### Detection and quantification of WYMV

Forty four collected wheat samples were tested for WYMV using both RT-qPCR and gel-based RT-PCR assay. Among the total of 44 samples, 21 samples were detected as positive by RT-qPCR assay, while only 11 samples were showed as positive by gel-based RT-PCR assay. Consistency between two assays for individual leaf tests was more than 57.1% (Table 2). This result showed that the RT-qPCR could yield more accurate, and detected more positive samples in low virus titer compared with conventional gel-based RT-PCR.

**Table 2:** Detection of WYMV from wheat samples by real-time RT-PCR and gel-based RT-PCR

**Collection sites**	**Date**	**Number of samples**	**Positive samples by real-time RT-PCR**	**Positive samples by gel-based RT-PCR**	**Consistency (%)**^**a**^
Zhumadian	2012	10	6	4	4/6 (66.6)
Yangzhou	2012	10	4	2	2/4 (50)
Yantai	2012	10	4	1	1/4 (25)
Nanjing	2011	10	3	1	1/3 (33.3)
Huangchuan	2004	1	1	1	1/1 (100)
Jiangyan	2006	1	1	1	1/1 (100)
Zhouzhi	2007	1	1	1	1/1 (100)
Xiaqiao	2011	1	1	1	1/1 (100)
Total		44	21	12	12/21 (57.1)

^a^ Results of individual samples diagnosed by real-time RT-PCR and gel-based RT-PCR were compared.

**Figure 3:**
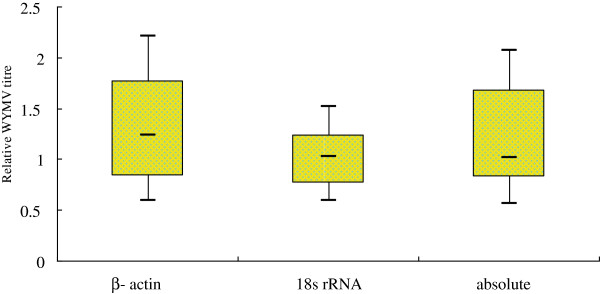
Determination of relative expression of WYMV using wheat β-actin gene and 18S rRNA as internal reference, and by absolute quantification with cloned standards (absolute).

Two different virus titers, the absolute and relative quantitation of all collected field samples, were carried out with cloned standard and reference genes (wheat β-actin gene and 18S rRNA). The results of WYMV content in infected wheat plants were ranged from 21.1 pg to 648 ng/100 mg wheat tissue when total RNA was used as template. The trends of the two reference genes were slightly different (Figure 3). The 18S rRNA assay resulted in low variation for all collected field samples, but the wheat β-actin gene and absolute quantitation results might lead to more variation. It was indicated that the wheat β-actin gene was not a good reference gene for valid experimental data. Absolute quantitation with cloned standards was inadequate to quantify the virus content in collected field samples. These results supported the necessity of the correct choice of reference genes for valid experimental data.

**Figure 4:**
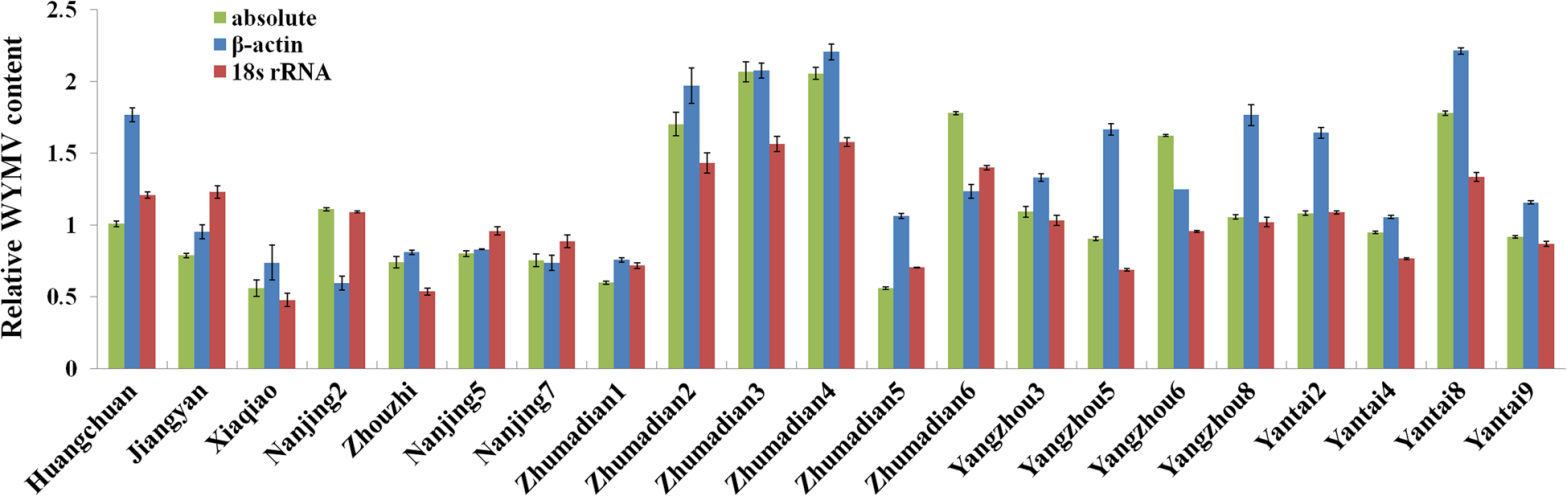
Determination of relative expression of WYMV in the 21 positive field samples determined by relative quantification wheat β-actin gene and 18S rRNA, and by absolute quantification with cloned standards (absolute).

The relative WYMV titer levels in the 21 positive field samples were determined by relative quantitation using the wheat β-actin gene and 18S rRNA as reference genes, and by absolute quantitation with cloned standards respectively (Figure 4). Results showed a higher virus titre in the samples collected from Huangchuan, Jiangyan and Zhumadian in 2012, where has been reported as serious epidemics of wheat yellow mosaic disease since the mid-1970s [5,6]. WYMV can survive to a long term in plasmodiophoral fungus, repeatedly infects the wheat seedlings and results in a higher virus titre [25].

## Discussion

The wheat diseases caused by WYMV have been responsible for significant economic loss in recent years in China since 1970 [2]. Diseases caused by WYMV were difficult to distinguish from other wheat virus disease because of their similar symptom. Now RT-PCR, RT-LAMP, and serological methods are most commonly used for detecting plant viruses, but by none of these methods it is possible to differentiate these wheat viruses precisely. In this study, a one step RT-qPCR assay was established for the detection, discrimination, and quantification of WYMV in wheat plants. This method could detect quantitatively WYMV in a short time and got a more sensitive result by using fluorescence signal.

The development of the TaqMan RT-PCR assays represents a significant advance in nucleic acid quantification. This approach utilizes the 5′ – 3′ exonuclease activity of *Thermus aquaticus* (Taq) DNA polymerase to cleave a dual-labeled probe annealed to a target sequence during amplification. The release of a fluorogenic tag from the 5′ end of the probe is proportional to the target sequence concentration or copy number, and then the increase in emission intensity can be monitored at each cycle in real-time [21]. The titer of *Rice stripe virus* and *Wheat dwarf virus* in field samples have been reported as few as 10 copies [22,23]. A minimum titer of *Tomato spotted wilt virus* was as less as 50 fg/100 mg [21]. In this assay, the virus titre of WYDV infected field samples were ranged from 21.1 pg to 648 ng/100 mg, similarly with above studies. The high virus titer was detected in the samples collected from the regions where wheat yellow mosaic disease has seriously epidemics for long time.

In RT-qPCR assay, the Ct value is a parameter reflecting the quantity of template presented in the reaction [18,22,26]. Usually, lower Ct values indicate a high concentration of template and higher Ct values indicate a low concentration of template [27]. This study showed that standard serial dilution curve was obtained with high RT-qPCR efficiency for WYMV (105.5%). The efficiency proved the balance among enzyme, dNTPs, primers and templates. The rationality of linear equation was determined by the coefficient of determination (R^2). Usually, R^2 value needs to be between 0 and 1, higher value indicates its higher rationality. In this study, the R^2 values of WYMV was 0.989. We therefore suggested that this RT-qPCR could be used to detect quantitatively WYMV in routine virus diagnosis.

The appropriate application of RT-qPCR, however, requires the use of reference genes whose level of expression is not affected by the test, general physiological conditions or inter-individual variability. RNA extraction errors, template loading deviations, and variations of reverse transcription efficiency could be eliminated from data analysis by quantification of the reference genes. The present study established a RT-qPCR assay that can be used for detection and absolute quantification of WYMV in wheat tissues. Wheat β-actin gene and 18S rRNA were selected as internal controls for their stability across all the tissues examined in wheat [28]. In this assay, there were some difference in stability between the two internal controls, 18s RNA were more stable than β-actin, which proved the necessity for choosing two internal controls.

Comparing with ELISA and gel-based RT-PCR, this assay has its advantages, i.e. not needing specific antibodies, spending less time (2–3 hours only) and having more sensitivity. The decrease of threshold for virus detection leads to an improvement of control schemes for plant virus diseases, especially for perennial soil-borne diseases. So, this quantitative detection of WYMV could be a useful method for epidemiological studying and forecasting and controlling the diseases.

## Conclusion

In conclusion, a sensitive and specific real-time RT-PCR method was developed and evaluated for WYMV detection of collected wheat samples. Compared with conventional gel-based RT-PCR, RT-qPCR yielded more accurate results, and detected the samples at low virus titer. According to above mentioned results, this RT-qPCR assay is robust and suitable for identification of the large numbers of field primary hosts and alternative hosts such as weeds. This method will provide as a potential application in early diagnosis, screening of resistant wheat varieties and identification of virus reservoirs.

## Materials and methods

### Plant material and virus sources

WYMV isolates collected from Nanjing in 2011 were detected by RT-PCR using primers targeting the WYMV CP gene and sequencing analysis [11]. Forty-four field wheat samples showing extreme chlorotic streak or mosaic even necrotic spots were respectively collected from Huangchuang, Jiangyan, Xiaqiao, Nanjing, Zhumadian, Yangzhou, Zhouzhi and Yantai in the years of 2004, 2006, 2007, 2011 and 2012, and stored at −70°C. The polyclonal antibody against WYMV was prepared by expressed CP in *Escherichia coli*, and then purified CP used as antigens to inject rabbit. The goat anti-rabbit IgG (H+L) alkaline phosphatase was supplied by Promega (Madison, WI, USA).

### RNA extraction

Total RNA from wheat leaf (stored at −70°C) was extracted using the RNAiso Reagent (TaKaRa Biotech., Dalian, China) according to the manufacturer’s instruction: grind the 0.1 g of leaf with the liquid nitrogen in the mortar, add 1ml Trizol reagent to the power, put them at the normal temperature for 5 min; transform the liquid to the 1.5 ml tube, add 200 μl chloroform was added, the tubes were shaken for 15 s, lay aside 15 min at −20°C; centrifuged at 12000 rpm for 15 min; the supernatant was transferred into a new tube, equal amount of isopropanol was added to the supernatant and the samples were put at normal temperature about 30 min; centrifuged at 12,000 rpm for 15 min; the pellet was washed using 1ml of 75% ethanol; the ethanol was removed at Rapid Glass Dryer; 40 μl DEPC H2O were added and the samples were stored at −70°C.

The concentration of each RNA sample was measured with a NanoDrop ND-1000 spectrophotometer (NanoDrop Technologies, USA). Only the RNA samples with an A260/A280 ratio (an indication of protein contamination) of 1.9 – 2.1 and an A260/A230 ratio (an indication of reagent contamination) greater than 2.0 were used for the analysis. The integrity of RNA samples was assessed by agarose gel electrophoresis.

### Primers and probes design

Two different systems of primers and probes were designed to detect the WYMV (WYMV system) and internal control system (wheat housekeeping genes: wheat β-actin gene and 18S rRNA) by using by the PRIMER EXPRESS software (Applied Biosystems, Foster, CA, USA). For the detection of WYMV system, primers were designed to correspond with the 879 bp long full-length WYMV CP gene fragment. (GenBank accession number AJ130983). For fluorescence detection, a primer–probe combination was designed as WF (upstream), WP (TaqMan probe) and WR (downstream) targeting the conserved region within the WYMV CP gene. For detection of wheat housekeeping genes, two primer–probe combinations were designed as internal control: AF (upstream), AT (TaqMan probe) and AR (downstream) targeted the wheat β-actin gene [29], and SF (upstream), ST (TaqMan probe) and SR (downstream) targeted the wheat 18s rRNA gene (GenBank accession number BJ217595) [28]. All TaqMan probes were labeled with 6-carboxyfluorescein (FAM, excitation wavelength 494 nm, emission wavelength 521 nm) at the 5′-end, and carboxy-tetramethyl-rhodamine (TAMRA) at the 3′-end. The detail of all primers and probes were listed in Table 3.

**Table 3:** Primers and probes used for real time RT-PCR in this study

**Target**	**Accession number**	**Primer names**	**Sequence**	***Tm *(°C)**	**GC (%)**	**Nucleotide position**
CP full-length	AJ130983	WCP-F:	5′GCAGCTGACACACAAAC 3′	41	53	1156–1172
		WCP-R:	5′GGTTAGTTCTGGGTGTCC 3′	43	56	2017–2034
CP probe	AJ130983	WF:	[FAM]-GCCGCGTACGCTTTTGACT-[TAMRA]	55	58	1843–1861
		WT:	[FAM]-CTTTGTCCCCCGCCCGTGG-[TAMRA]	64	74	1863–1881
		WR:	[FAM]-TTCGATGTCCTGTGGGTTCA-[TAMRA]	53	50	1883–1902
Wheat β-actin	AB181991	AF:	[FAM]-TCCAATCTATGAGGGATACACGC-[TAMRA]	55	48	263–286
		AT:	[FAM]-TTCCTCAAGCTATCCTTCGTTTGGACCTG-[TAMRA]	65	48	286–314
		AR:	[FAM]-TCTTCATTAGATTATCCGTGAGGTC-[TAMRA]	53	40	324–348
Wheat 18S rRNA	BJ217595	SF:	[FAM]-CAGGCGATGGGAAA-[TAMRA]	42	57	199–212
		ST:	[FAM]-TCGCTTTGAGTTTGGGCAATTGTGG-[TAMRA]	65	48	136–160
		SR:	[FAM]-CATTGGAGGGCAAGTCT-[TAMRA]	45	53	283–299

The resulting primers and probes are shown as follow: To the WYMV, the first pair of primers of WYMV: WYMV CP -F: 5′- GCAGCTGACACACAAAC -3′ (upstream, *T*m=41 °C) and WYMV CP-R: 5′- GGTTAGTTCTGGGTGTCC -3′ (downstream, *Tm*=43°C) correspond to the WYMV CP (GenBank accession number AJ130983), and were expected to amplify a fragment of 879bp for the standard sample. For fluorescence detection, one primer–probe combination was also selected from combinations proposed by the PRIMER EXPRESS software (Applied Biosystems, USA) according to the manufacturer’s instructions. The first primer–probe combination was designed: 1843F:5′- GCCGCGTACGCTTTTGACT -3′ (upstream, *Tm*=55°C), 1863T:5′- CTTTGTCCCCCGCCCGTGG -3′ (TaqMan probe, *Tm*=64°C), and 1883R:5′- TTCGATGTCCTGTGGGTTCA -3′ (downstream, *Tm*=53°C) targeting the conserved region within WYMV CP (GenBank accession number AJ130983). WYMV probe was labeled at the 5′end with 6-carboxyfluorescein (FAM, excitation wavelength 494 nm, emission wavelength 521 nm) at the 5′-end, and carboxy-tetramethyl-rhodamine (TAMRA) at the 3′-end.

For the wheat housekeeping gene, the sequence of the wheat β-actin gene (GenBank accession number AB181991) was selected. The primer–probe combination: 263F: 5′- TCCAATCTATGAGGGATACACGC - 3′ (upstream, *Tm*=55°C), 286T: 5′- TTCCTCAAGCTATCCTTCGTTTGGACCTG -3′ (TaqMan probe, *Tm*=65°C) and 324R: 5′- TCTTCATTAGATTATCCGTGAGGTC-3′ (downstream, *Tm*=53°C) was designed to target the wheat β-actin gene as an internal control. And to another housekeeping gene, the sequence of the wheat 18S rRNA (GenBank accession number BJ217595): the primer–probe combination: 199F: 5′- CAGGCGATGGGAAA-3′ (upstream, *Tm*=42°C), 136T: 5′- TCGCTTTGAGTTTGGGCAATTGTGG -3′ (TaqMan probe, *Tm*=65°C) and 283R: 5′- CATTGGAGGGCAAGTCT-3′ (downstream, *Tm*=45°C) was designed to target the wheat 18S rRNA as another internal control.

All TaqMan probes were labeled with 6-carboxyfluorescein (FAM, excitation wavelength 494 nm, emission wavelength 521 nm) at the 5′ end and carboxy-tetramethyl-rhodamine (TAMRA) at the 3′-end.

### Preparation for viral RNA standards of one-step RT-qPCR

RNA transcripts were synthesized in vitro, inserting the cDNA fragments of WYMV CP, β-actin and 18S rRNA into the pGEM T-EASY (Promega, USA) respectively, and then transforming them into competent cell of *Escherichia coli* strain JM110, The right inserted PCR products was monitored by gel electrophoresis of restriction enzyme cleavage.

Positive single strand RNA was transcribed using the RiboMAX Large Scale RNA Production Systems-T7 Kit (Promega, USA), using 2 μg linearized plasmid DNA as template, then treated by DNaseI at 37°C for 20 min, purifying the RNA by RNAclean kit (BioTeke, China). The purified RNA was quantified using NanoDrop ND-1000.

### One-step real time RT-PCR assay and optimization

One-step real-time RT-PCR assay was performed with the final volume of 20 μl using the One-step PrimeScript RT-PCR Kit (TaKaRa Biotech, Dalian, China) according to the manufacturer’s instructions. Each reaction was carried out using 2 μl of total RNA from collected samples on the Bio-Rad iCycler IQ Real-Time PCR Detection System. Data were analyzed with iCycler IQ Real-Time PCR Detection System Software Version 3.1.

Upstream and downstream primers were subjected to a 3 × 3 optimization matrix using a concentration of 100, 200 and 400 nM whereas the probe’s concentration used was 100 nM. RNA transcripts synthesized in vitro were used as template. The concentration of the TaqMan probe was then optimized in order to reduce the quantity used in reactions. The parameters of the reaction program were examined to determine the most suitable program. The most suitable program and parameter were reverse transcription of the viral RNA at 42°C for 5 min. PCR was performed with the hot-start *Taq* polymerase included an enzyme activation step (95°C for 5 s), followed by 45 cycles of denaturation/annealing–extension (10 s at 95°C; 60 s at 60°C). Gel-based PCR assay was carried out for 35 cycles, each consisting of denaturation at 94°C for 1 min, annealing at 65°C for 45 s, and extension at 72°C for 2 min, with 94°C for 2 min at the beginning and 72°C for 10 min at the final step. The expected PCR products were 879 bp, using the primer pairs of WCP-F: 5′-GCA GCT GAC ACA CAA AC-3′ (upstream) and WCP-R: 5′-GGT TAG TTC TGG GTG TCC-3′ (downstream). The PCR products were detected by 1.0% agarose gels electrophoresis.

Viral RNA transcripts (cloned standard), prepared as described above, were used in tenfold serial dilution to generate standard curves for determination of the assay efficiency. Virus content of collected field samples were analyzed by two different virus tittering: with clone standard for absolute quantiation and with housekeeping genes (wheat β-actin gene and 18S rRNA) for relative quantitation. The data were analyzed by one and two-way analysis of variance.

## Competing interests

The authors declare that they have no competing interests.

## Authors’ contributions

WL contributed to the design of the study, primer design, statistical analysis and designing the RT-qPCR protocol. XZ contributed to the RNA extractions, optimization of the RT-qPCR assay, screening of samples, statistical analysis, and drafting the manuscript. PZ contributed to the sample collection, RNA extraction and drafting of the manuscript. TTM contributed to the sample collection, RNA extraction and drafting of the manuscript. YL contributed to the design of the study, sample collection and drafting of the manuscript. ZZ contributed to the sample collection and optimization of the RT-qPCR assay. CH contributed to the design of the study and drafting of the manuscript. XW contributed to the design of the study, sample collection, data analysis and drafting the manuscript. All authors read and approved the final manuscript.

## References

[B1] Ordon F Habekuss A Kastirr U Rabenstein F Kühne T Virus resistance in cereals: sources of resistance, genetics and breeding J Phytopathol 2009 157 535 545 10.1111/j.1439-0434.2009.01540.x

[B2] Wang X Liu Y Han C Wu Y Zhao Z Present situation and development strategies for the research and control of wheat viral diseases Plant Protection 2010 36 13 19

[B3] Wu B Blanchard-Letort A Liu Y Zhou G Wang X Elena SF Dynamics of molecular evolution and phylogeography of Barley yellow dwarf virus-PAV PLoS One 2011 6 e16896 10.1371/journal.pone.0016896 21326861 PMC3033904

[B4] Liu Y Zhai H Zhao K Wu B Wang X Two suppressors of RNA silencing encoded by cereal-infecting Luteoviridae J Gen Virol 2012 93 1825 1830 10.1099/vir.0.042135-0 22592264

[B5] Chen J Ocurrence of fungally transmitted wheat mosaic viruses in China Ann Appl Biol 1993 123 55 61 10.1111/j.1744-7348.1993.tb04072.x

[B6] Han C Li D Xing Y Zhu K Tian Z Cai Z Yu J Liu Y Wheat yellow mosaic virus widely occurring in wheat (*Triticum aestivum*) in China Plant Dis 2000 84 627 630 10.1094/PDIS.2000.84.6.627 30841101

[B7] Hariri D Courtillot M Zaoui P Lapierre H Multiplication of soilborne wheat mosaic virus (SBWMV) in wheat roots infected by a soil carrying SBWMV and wheat yellow mosaic virus Agronomie 1987 7 789 796 10.1051/agro:19871005

[B8] Lin M Ruan Y A study on wheat yellow mosaic virus Acta Phytopathol Sin 1986 16 73 78

[B9] Slykhuis JT Barr DJS Confirmation of polymyxa graminis as a vector of wheat spindle streak mosaic virus Phytopathology 1978 68 639 643 10.1094/Phyto-68-639

[B10] Usugi T Saito Y Relationships between wheat yellow mosaic virus and wheat spindle streak mosaic virus Ann Phytopathol Soc Jpn 1979 45 397 400 10.3186/jjphytopath.45.397

[B11] Chen J Sohn A Chen JP Lei J Cheng Y Schulze S Steinbiss HH Antoniw JF Adams MJ Molecular comparisons amongst wheat bymovirus isolates from Asia, North America and Europe Plant Pathol 1999 48 642 647 10.1046/j.1365-3059.1999.00392.x

[B12] Yu J Yan L Su N Huo Z Li D Han C Yang L Cai Z Liu Y Analysis of nucleotide sequence of wheat yellow mosaic virus genomic RNAs Sci China (Ser C) 1999 42 554 560 10.1007/BF02881780 18726520

[B13] Li D Yan L Su N Han C Hou Z Yu J Liu Y The nucleotide sequence of a Chinese isolate of wheat yellow mosaic virus and its comparison with a Japanese isolate Arch Virol 1999 144 2201 2206 10.1007/s007050050633 10603173

[B14] Namba S Kashiwazaki S Lu X Tamura M Tsuchizaki T Complete nucleotide sequence of wheat yellow mosaic bymovirus genomic RNAs Arch Virol 1998 143 631 643 10.1007/s007050050319 9638137

[B15] Hariri D Delaunay T Gomes L Filleur S Plovie C Lapierre H Comparison and differentiation of wheat yellow mosaic virus (WYMV), wheat spindle streak mosaic virus (WSSMV) and barley yellow mosaic virus (BaYMV) isolates using monoclonal antibodies Eur J Plant Pathol 1996 102 283 292 10.1007/BF01877967

[B16] Clover G Henry C Detection and discrimination of wheat spindle streak mosaic virus and wheat yellow mosaic virus using multiplex RT-PCR Eur J Plant Pathol 1999 105 891 896 10.1023/A:1008707331487

[B17] Zhang Z Liu X Li D Yu J Han C Rapid detection of wheat yellow mosaic virus by reverse transcription loop-mediated isothermal amplification Virol J 2011 8 550 10.1186/1743-422X-8-550 22185375 PMC3260119

[B18] Boonham N Smith P Walsh K Tame J Morris J Spence N Bennison J Barker I The detection of Tomato spotted wilt virus (TSWV) in individual thrips using real-time fluorescent RT-PCR (TaqMan) J Virol Methods 2002 101 37 48 10.1016/S0166-0934(01)00418-9 11849682

[B19] Giovanna M Piero C Gian P Emanuela N Real-time PCR for the quantitation of tomato yellow leaf curl Sardinia virus in tomato plants and in *bemisia tabaci* J Virol Methods 2008 147 282 289 10.1016/j.jviromet.2007.09.015 17980920

[B20] Lopez R Asensio C Guzman MM Boonham N Development of real-time and conventional RT-PCR assays for the detection of potato yellow vein virus (PYVV) J Virol Methods 2006 136 24 29 10.1016/j.jviromet.2006.03.026 16712962

[B21] Roberts CA Dietzgen RG Heelan LA Maclean DJ Real-time RT-PCR fluorescent detection of tomato spotted wilt virus J Virol Methods 2000 88 1 8 10.1016/S0166-0934(00)00156-7 10921836

[B22] Zhang X Wang X Zhou G A one-step real time RT-PCR assay for quantifying rice stripe virus (RSV) in rice and in the small brown planthopper (*Laodelphax striatellus* Fallen) J Virol Methods 2008 151 181 187 10.1016/j.jviromet.2008.05.024 18586332

[B23] Zhang X Zhou G Wang X Detection of Wheat dwarf virus (WDV) in wheat and vector leafhopper (*Psammotetix striatus* L.) by real-time PCR J Virol Methods 2010 169 416 419 10.1016/j.jviromet.2010.07.029 20691208

[B24] Zhang P Mar T Liu W Li L Wang X Simultaneous detection and differentiation of Rice black streaked dwarf virus (RBSDV) and Southern rice black streaked dwarf virus (SRBSDV) by duplex real time RT-PCR Virol J 2013 10 24 10.1186/1743-422X-10-24 23331990 PMC3610162

[B25] Jianping C Wilson TMA Taxonomy of rigid rod-shaped viruses transmitted by fungi Agronomie 1995 15 421 426 10.1051/agro:19950706

[B26] Gadiou S Ripl J Jaňourová B Jarošová J Kundu JK Real-time PCR assay for the discrimination and quantification of wheat and barley strains of *Wheat dwarf virus* Virus Genes 2012 44 349 355 10.1007/s11262-011-0699-0 22173982

[B27] Ruiz–Ruiz S Ambrós S Vives MC Navarro L Moreno P Guerri J Detection and quantitation of *Citrus leaf blotch virus* by TaqMan real-time RT-PCR J Virol Methods 2009 160 57 62 10.1016/j.jviromet.2009.04.012 19406167

[B28] Jarošová J Kundu JK Validation of reference genes as internal control for studying viral infections in cereals by quantitative real-time RT-PCR BMC Plant Biol 2010 10 146 10.1186/1471-2229-10-146 20630112 PMC3095291

[B29] Li X Zhang JB Song B Li HP Xu HQ Qu B Dang FJ Liao YC Resistance to Fusarium head blight and seedling blight in wheat is associated with activation of a cytochrome P450 gene Phytopathology 2010 100 183 191 10.1094/PHYTO-100-2-0183 20055652

